# The K^+^-Dependent and -Independent Pyruvate Kinases Acquire the Active Conformation by Different Mechanisms

**DOI:** 10.3390/ijms23031347

**Published:** 2022-01-25

**Authors:** Leticia Ramírez-Silva, Gloria Hernández-Alcántara, Carlos Guerrero-Mendiola, Martin González-Andrade, Adela Rodríguez-Romero, Annia Rodríguez-Hernández, Alan Lugo-Munguía, Paul A. Gómez-Coronado, Cristina Rodríguez-Méndez, Alicia Vega-Segura

**Affiliations:** 1 Departamento de Bioquímica, Facultad de Medicina, Apartado Postal 70-159, Universidad Nacional Autónoma de México, Mexico City 04510, Mexico; ghernandez@bq.unam.mx (G.H.-A.); martin@bq.unam.mx (M.G.-A.); alanlugo117@gmail.com (A.L.-M.); Paul.Gomez@mpi-marburg.mpg.de (P.A.G.-C.); Cris_romen@outlook.com (C.R.-M.); malivega@hotmail.com (A.V.-S.); 2 Departamento de Genética Molecular, Instituto de Fisiología Celular, Universidad Nacional Autónoma de México, Mexico City 04510, Mexico; carlos@bq.unam.mx; 3 LANEM, Instituto de Química, Universidad Nacional Autónoma de México, Mexico City 04510, Mexico; adela@unam.mx (A.R.-R.); arodriguezh@iquimica.unam.mx (A.R.-H.)

**Keywords:** pyruvate kinase, enzyme purification, enzyme kinetics, crystal structure, molecular dynamics, circular dichroism, K^+^-dependent pyruvate kinase, K^+^-independent pyruvate kinase

## Abstract

Eukarya pyruvate kinases possess glutamate at position 117 (numbering of rabbit muscle enzyme), whereas bacteria have either glutamate or lysine. Those with E117 are K^+^-dependent, whereas those with K117 are K^+^-independent. In a phylogenetic tree, 80% of the sequences with E117 are occupied by T113/K114/T120 and 77% of those with K117 possess L113/Q114/(L,I,V)120. This work aims to understand these residues’ contribution to the K^+^-independent pyruvate kinases using the K^+^-dependent rabbit muscle enzyme. Residues 117 and 120 are crucial in the differences between the K^+^-dependent and -independent mutants. K^+^-independent activity increased with L113 and Q114 to K117, but L120 induced structural differences that inactivated the enzyme. T120 appears to be key in folding the protein and closure of the lid of the active site to acquire its active conformation in the K^+^-dependent enzymes. E117K mutant was K^+^-independent and the enzyme acquired the active conformation by a different mechanism. In the K^+^-independent apoenzyme of *Mycobacterium tuberculosis*, K72 (K117) flips out of the active site; in the holoenzyme, K72 faces toward the active site bridging the substrates through water molecules. The results provide evidence that two different mechanisms have evolved for the catalysis of this reaction.

## 1. Introduction

Rabbit muscle pyruvate kinase (WT-RMPK) was the first demonstration of an enzyme that requires monovalent cations for catalysis [1]. WT-RMPK exhibits an activity of 250 and 0.02 μmoles min^−1^mg^−1^ with and without 90 mM K^+^ [2], which can be partially substituted with Rb^+^, NH_4_^+,^ and Tl^+^, albeit with activity 20–40% lower. In the presence of Cs^+^, Na^+^ and Li^+^, the enzyme exhibits only 9%, 8%, and 2%, respectively, of the maximal activity and hardly any activity (0.02%) with tris-(hydroxymethyl) aminomethane (Tris) [3] or (<0.001%) with substituted amines [4].

For many years, it was assumed that all pyruvate kinases (PKs) had an absolute requirement for K^+^, until 1969 when Benziman reported that the PK from *Acetobacter xylinium* did not require monovalent cations for activity [5]. After this first report, other examples of K^+^-independent enzymes arose [6,7,8,9,10,11,12,13,14]. In this respect, Laughlin and Reed, 1997, compared the sequences of the PKs from *E. coli* Type II, *C. glutamicum* and WT-RMPK [15]. The authors found that E117 (numbered according to WT-RMPK residues), close to the K^+^ binding site, has been substituted for Lys in the microbial enzymes. The proximity of the side chain from residue 117 to the binding site of K^+^ in WT-RMPK suggested that in the monovalent cation-independent enzymes, ε-NH_3_^+^ of Lys provides the inner positive charge that substitutes the activating effect of K^+^. Therefore, the authors constructed the mutant E117K and found it was K^+^-independent with ~12% of the maximal activity of the wild type enzyme with K^+^. Furthermore, it appeared that the activity was not stimulated by monovalent cations [15].

**Figure 1:**
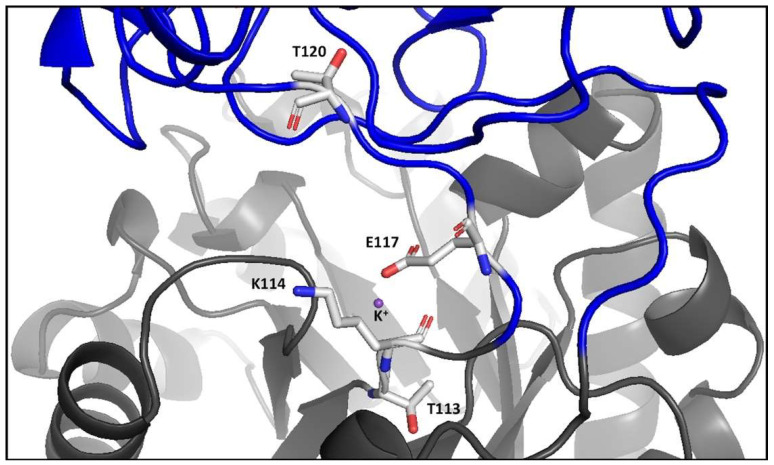
Ribbon representation of the boundary region between domains A (gray) and B (blue) of WT-RMPK. According to Oria-Hernández et al. (2006) [16], short loop residues T113, K114, E117, and T120 co-evolve in a consensus sequence in the family of the K^+^-dependent PKs in 80% of the 121 sequences; whereas L113, Q114, K117 and (L, V, I) 120 co-evolve in the K^+^-independent PKs in 77% of the 106 sequences. Residues of RMPK are illustrated in sticks and K^+^ in purple. The figure was constructed with PDB 1A49, subunit A.

An extensive phylogenetic study of the PK family was conducted to establish how frequently E117 is replaced by K117 (according to the numbering of WT-RMPK) [16]. This analysis showed two clearly separated clusters of roughly the same number of sequences, one with E117 and the other with K117. All characterized enzymes with E117 exhibit K^+^-dependent activity, whereas those with K117 are K^+^-independent. Another relevant feature consists in the apparent co-evolution between the residue present at position 117 and those at positions 113, 114, and 120: 80% of the sequences that have E117 are occupied by T113/K114/T120, and 77% of those that have K117 possess L113/Q114/ (L, I, V)120. As shown in Figure 1, residues 114–118 form a short loop that connects the A and B domains. Residues 113 and 120 lie at the beginning and end of the loop, respectively. The active site sits in a cleft formed by these two domains [17].

This work focuses on understanding the individual and combined roles of highly conserved residues forming part of a ’signature’ sequence of K^+^-independent PKs. To this end, the residues at these positions in the K^+^-dependent sequence of WT-RMPK (T113/K114/E117/T120) were gradually replaced by those from K^+^-independent PKs (L113/Q114/K117/L120). It was expected that the quadruple mutant T113L/K114Q/E117K/T120L would confer K^+^-independence and would exhibit activities like those of WT-RMPK with K^+^. However, results indicate that K^+^-independent activity increases with the addition of residues L113 and Q114 to K117, obtaining half of the expected activity; but the addition of residue L120 leads to a nearly inactive enzyme. The secondary structure of mutants possessing T120L exhibited anomalous circular dichroism (CD) ellipticities compared to those of WT-RMPK and the other mutants, indicating different foldings. T120 and L120 play different roles; with the former related to the folding and closure of the lid in the K^+^-dependent enzymes. On the other hand, K117 or K72 (as found in *Mycobacterium tuberculosis* (*Mtb*PK)) not only conferred K^+^-independence to PKs, but appeared key in the acquisition of the active conformation of the K^+^-independent PKs as demonstrated by the comparison of crystallographic data and molecular dynamic (MD) analysis of WT-RMPK (K^+^-dependent) and those of the *Mtb*PK (K^+^-independent) [18]. Overall data provide convincing evidence that two different mechanisms have evolved for the catalysis of this reaction. 

## 2. Results and Discussion

### 2.1. Effect of Li^+^, Na^+^, K^+^, NH_4_^+^, Rb^+^, Cs^+^, (CH_3_)_4_N^+^ and (CH_3_)_3_N^+^-CH_2_-CH_2_-OH (Choline) on the Activities of WT-RMPK and RMPK Mutants 

**Figure 2:**
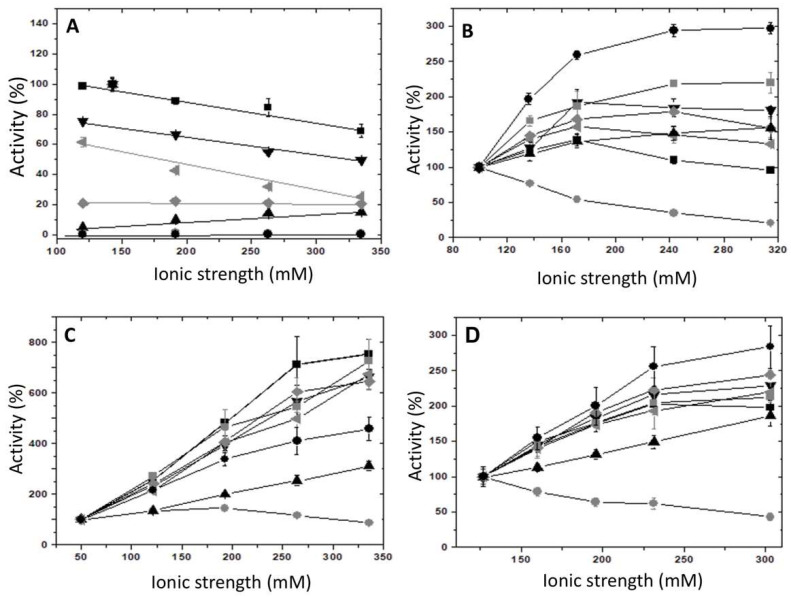
Effect of ionic strength on the activities of WT-RMPK (**A**), E117K (**B**), T113L/E117K (**C**), and T113L/K114Q/E117K (**D**) mutants induced by the indicated monovalent cations. In the absence of monovalent cations, the reaction mixtures contained ionic strengths of 99.5, 50, and 126.8 mM in (**B**–**D**), respectively. The activities in these conditions for (**B**–**D**) were 14 ± 3, 10 ± 1 and 34 ± 4, respectively. In the presence of 90 mM K^+^, the activity for (**A**) was 263 ± 15 and ionic strength 143 mM. These activities were normalized to 100%, which corresponded to the control activity for each PK. Besides 25 mM HEPES-(CH_3_)_4_NOH pH 7.4, 0.2 mM NADH, 8 μg/ml LDH and a reaction mixture with saturating concentrations of substrates, Li^+^ (●), Na^+^ (▲), K^+^ (∎), NH_4_^+^ (◀), Rb^+^ (▼), Cs^+^ (♦), choline (●) or (CH_3_)_4_N^+^ (∎) were added to complete the ionic strengths indicated in the figure. Reaction mixtures contained: (**A**) 0.64 mM PEP^3−^, 2.5 mM Mg-ADP, 1.69 mM Mg^2+^_free_; (**B**) 0.63 mM PEP^3−^, 2 mM Mg-ADP and 19 mM Mg^2+^_free_; (**C**) 0.54 mM PEP^3−^, 0.7 mM Mg-ADP and 3 mM Mg^2+^_free_ and (**D**) 0.67 mM PEP^3−^, 1.84 mM Mg-ADP and 24 mM Mg^2+^_free_. Assays were performed at 25 °C, and the reaction was started by the addition of PK. Amounts of PK ranged from 0.1 to 0.2 μg/mL. Standard deviation bars of three experiments are shown.

According to previous studies [15], it was expected that mutants of RMPK with Lys in position 117 would exhibit K^+^-independent activity. To explore the activation of these enzymes by monovalent cations, specific effects first need to be distinguished from non-specific ionic strength effects. To this end, the effect of monovalent cations such as Li^+^, Na^+^, K^+^, NH_4_^+^, Rb^+^, Cs^+^, and that of bulky “inert” monovalent cations like (CH_3_)_4_NC^+^ and choline were studied on desalted WT-RMPK, E117K, T113L/E117K, and T113L/K114Q/E117K mutants. K114Q/E117K/T120L mutant was completely inactive and T120L and T113L/K114Q/E117K/T120L mutants were nearly inactive. Therefore, they were not included in the next experiments. (CH_3_)_4_N^+^ and choline are commonly used to maintain ionic strength [19] because either they do not interact with RMPK or they interact at non-specific sites on the protein [4]. An inhibitory effect due to high ionic strength (200 mM) has also been observed in RMPK with either (CH_3_)_4_N^+^ or activating monovalent cations [4]. Consequently, the effect of these bulky monovalent cations ((CH_3_)_4_N^+^ or choline) on WT-RMPK observed in Figure 2A was expected (less than 0.01% of maximal activity). Maximal activities were shown with 90 mM K^+^ (control) followed by Rb^+^ > NH_4_^+^ > Cs^+^ > Na^+^ > Li^+^. These activities also decreased with increasing ionic strength over 200 mM. In contrast, E117K (Figure 2B), T113L/E117K (Figure 2C), and T113L/K114Q/E117K mutants (Figure 2D) were 3, 7, and 2.7-fold activated by ~250 mM choline or (CH_3_)_4_N^+^, respectively. Furthermore, activating monovalent cations increased the activities of these three RMPK mutants. 

**Figure 3:**
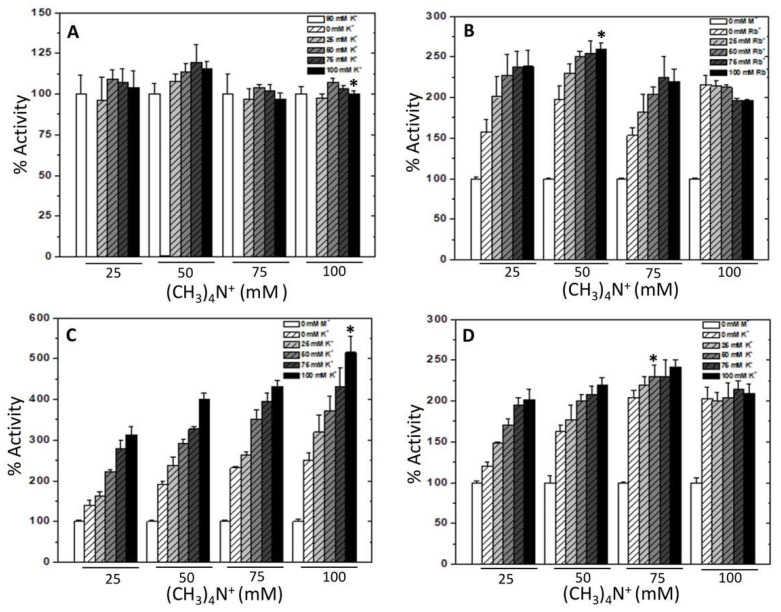
Effect of the addition of various concentrations of K^+^ (or Rb^+^) and (CH_3_)_4_N^+^ on the activities of WT-RMPK (**A**), E117K (**B**), T113L/E117K (**C**) and T113L/K114Q/E117K (**D**) mutants. Ionic strength and activities with 90 mM K^+^ (**A**) and without monovalent cations (**B**–**D**) were those indicated in the legend of Figure 2. The normalized control activities were shown in white columns in (**A**–**D**). White striped columns were in the presence of 25, 50, 75, and 100 mM (CH_3_)_4_N^+^ concentrations without K^+^ (or Rb^+^). In addition to the indicated (CH_3_)_4_N^+^ concentration, light gray, gray, dark gray, and black striped columns were in the presence of the 25, 50, 75, and 100 mM K^+^ (or Rb^+^) concentrations, respectively. The highest activities of RMPK mutants were achieved with ionic strength of 250 mM pointed out with *. Standard deviation bars of three experiments are shown.

The effects described herein were general for tetraalkylammonium salts, and monovalent cations and activities increased with ionic strength, except with Li^+^. Therefore, the effect over E117K, T113L/E117K, and T113L/K114Q/E117K mutants was due to a generalized ionic strength effect. Next, it was investigated whether the ionic strength effect of bulky and activating monovalent cations was additive. As expected, WT-RMPK (Figure 3A) expressed high activities with 90 mM K^+^ (white column) versus no activity without K^+^ and up to 100 mM (CH_3_)_4_N^+^ (white striped columns, not seen due to the scale). Activities were observed with 25 mM K^+^ and increased slightly with increasing K^+^ concentration but remained largely the same with different concentrations of (CH_3_)_4_N^+^. Hence, no additive effect was observed between K^+^ and bulky monovalent cation. In contrast, K^+^-independent activities (white columns) of E117K (Figure 3B), T113L/E117K (Figure 3C), and T113L/K114Q/E117K mutants (Figure 3D) were gradually raised by either the addition of an increasing amount of (CH_3_)_4_N^+^ (white striped columns) or of the bulky cation plus K^+^ or Rb^+^ (gray colored striped columns). That is to say that, the activating effect of bulky and monovalent cations were additive. Maximal activities of mutant PKs were achieved with 250 mM ionic strength, indicated with asterisks. Consequently, the next experiments were all performed in the presence of (CH_3_)_4_N^+^ to maintain constant ionic strength at 250 mM. 

In previous studies of the E117K mutant [15,20], this effect was not observed because both used (CH_3_)_4_N^+^ to maintain ionic strength constant. Still, they did not explore the effect of increasing ionic strength on the activity of this mutant. Following the phylogenetic analysis [16], K^+^ binds to a highly conserved site. In the K^+^-independent PKs, three of the four residues that coordinate K^+^ are conserved in most enzymes. The only residue missing is T113 (numbering according to RMPK) which is Leu in K^+^-independent PKs. Hence, it is not surprising that these PKs still bind a monovalent cation as observed in the structure of *Mtb*PK [18].

### 2.2. Effect of Activating Monovalent Cations to WT-RMPK and RMPK Mutants in the Presence of 250 mM Ionic Strength Maintained with (CH_3_)_4_N^+^

**Figure 4:**
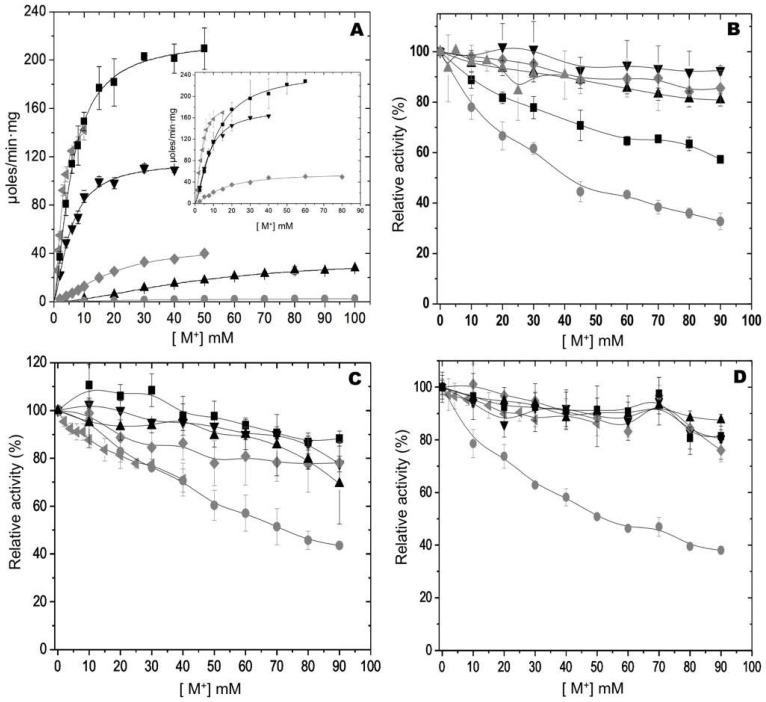
Effect of monovalent cations on the activities of WT-RMPK (**A**), K114Q (inset of A), E117K (**B**), T113L/E117K (**C**), and T113L/K114Q/E117K (**D**) mutants. The activities of WT-RMPK (**A**) and K114Q mutant (inset of A) and of the normalized activities of E117K (**B**), T113L/E117K (**C**) and T113L/K114Q/E117K (**D**) mutants in the presence of the indicated concentrations of Li^+^ (●), Na^+^ (▲), K^+^ (■), NH_4_^+^ (◀), Rb^+^ (▼) or Cs^+^ (♦) are shown. In the absence of monovalent cations and ionic strength of 250 mM, the activities of (**B**–**D**) were 36 ± 6, 51 ± 1 and 82 ± 7, respectively. The experimental conditions were as in Figure 2, except that the ionic strength was maintained constant at 250 mM with (CH_3_)_4_N^+^. The reaction mixture for K114Q mutant, contained 1.5 mM PEP^3−^, 5.4 mM Mg-ADP and 3 mM Mg^2+^_free_ and 135 mM of ionic strength. Standard deviation bars of three experiments are shown.

The activation of WT-RMPK, K114Q, E117K, T113L/E117K and T113L/K114Q/E117K mutants by different concentrations of Li^+^, Na^+^, K^+^, NH_4_^+^, Rb^+^ and Cs^+^ were measured and compared in the presence of saturating concentrations of PEP^3−^, Mg-ADP complex and Mg^2+^_free_ and constant ionic strength of 250 mM (Figure 4). In confirmation of previous results [3], it was observed that maximal WT-RMPK activation followed the order K^+^ > NH_4_^+^ > Rb^+^ > Cs^+^ > Na^+^ > Li^+^. It is important to note that the activation curves of WT-RMPK with Na^+^ and Li^+^ did saturate (see Table 1). In previous studies, WT-RMPK activation exhibited no saturation of these cations at an ionic strength of 223 mM ionic strength maintained with HEPES [21]. (CH_3_)_4_N^+^ may bind to non-specific sites of the enzyme favoring Na^+^ and Li^+^ occupation of the monovalent binding site. As expected K114Q mutant was K^+^-dependent. Ion selectivity was preserved and kinetic constants for the monovalent cations were as those of wild-type PK (Table 1). As reported for RMPK [22,23], it is expected that K114Q follow Type Ib activation by monovalent cations. In such a mechanism, M^+^ coordination is absolutely required for catalysis, where both *k_cat_* and *k_cat_*/*K_m_* increase hyperbolically with K^+^. In contrast, ion selectivity of T113L was altered; maximal activation followed the order Rb^+^ > NH_4_^+^ > K^+^ > Cs^+^. It was found that the polarity of residue 113 is determinant in the partition of K^+^ into its site [21]. E117K (Figure 4B), T113L/E117K (Figure 4C), and T113L/K114Q/E117K mutants (Figure 4D) were K^+^-independent. In the presence of 250 mM ionic strength maintained with (CH_3_)_4_N^+^, no activation was further found for these mutants in the presence of the monovalent cations, only inhibition; i.e., their activities with 90 mM Li^+^ was 40% of that obtained without this cation. 

### 2.3. Kinetics of WT-RMPK and RMPK Mutants 

The K^+^-dependent PKs signature (T113/K114/E117/T120) was gradually substituted for that of the K^+^-independent PKs (L113/Q114/K117/L120). It was expected that the quadruple mutant T113L/K114Q/E117K/T120L would be K^+^-independent and would express a similar activity to that of WT-RMPK with K^+^. In this respect, it was observed that those mutants containing the substitution of Thr 120 for Leu became completely or nearly completely inactive after the last purification step. Therefore, the kinetic constants of WT-RMPK and all the mutants are shown in Table 2 except those containing the mutation T120L. Single mutants with E117 showed values of *k_cat_* similar to that of WT-RMPK; whereas the gradual change of the K^+^-independent signature to WT-RMPK (E117K, T113L/E117K, T113L/K114Q/E117K) followed a gradual increase in *k_cat_* from 35% to ~half catalytic constant of WT-RMPK with K^+^. In spite of the differences in catalytic constants, affinities for PEP^3−^ and Mg-ADP for mutants with E117 or K117 were similar to those of WT-RMPK with K^+^; whereas mutants with K117 exhibited Km for Mg^2+^_free_ from ~10 to 50-fold greater than that of WT-RMPK (*K*_Mg_^2+^_free_ 0.17 ± 0.01). 

It was consistently observed that K^+^-independent mutants exhibited very low affinities for Mg^2+^_free_ compared to K^+^-dependent PKs in this study and elsewhere [24]. This result may be explained by the presence of water molecules through which Mg-ATP coordinates to Mg^2+^ and oxalate in the *Mtb*PK structure [18], instead of direct coordination between substrates as in WT-RMPK structure [17]. The lower binding energy of Mg^2+^ could result from the stabilizing contribution to maintaining water molecules between the divalent cation and the other substrates.

These results indicate that K117 is responsible for the RMPK´s K^+^-independent activity. The addition of residues L113 and Q114 present in the K^+^-independent sequences, promotes an increase in the K^+^-independent activity and the dependence of high concentrations of Mg^2+^. Therefore, residues L113, Q114, and K117 can allow K^+^-independent activity in the RMPK, but the inclusion of L120 seems to impair the enzyme.

### 2.4. Circular Dichroism Spectra of WT-RMPK and RMPK Mutants 

**Figure 5:**
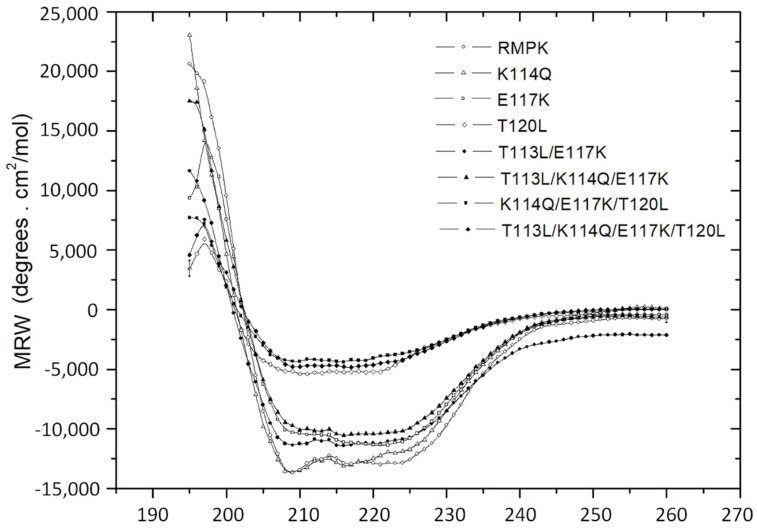
Far-UV CD spectra of WT-RMPK (○) and of the RMPK mutants: E117K (□), K114Q (△), T113L/E117K (●), T113L/K114Q/E117K (■), T120L (▽), K114Q/E117K/T120L (▲) and T113L/K114Q/E117K/T120L (▼). The spectra were obtained in mixtures that contained each pyruvate kinase at a concentration of 100 μg/mL in 25 mM phosphate buffer pH 7.0 at 25 °C in cell pathway of 0.1 cm. CD is expressed as θ_MRW_ (mean residual weight ellipticity).

To explore the effect on WT-RMPK structure of including residues observed in K^+^-independent PKs, we obtained the far UV circular dichroism (CD) spectra of WT-RMPK and all mutants. CD is widely used to determine whether an expressed recombinant purified protein is folded or if a mutation affects its conformation or stability [25]. Figure 5 shows that the spectra of WT-RMPK and K114Q almost overlapped; also a small loss of CD signal of ~15% to 20% was observed in E117K, T113L/E117K and T113L/K114Q/E117K mutants and a significative loss of CD signal of ~60% to 75% was observed for T120L, K114Q/E117K/T120L and T113L/K114Q/E 117K/T120L mutants. 

While most mutants are native-like, CD data also confirmed the absence of integrity of those containing the substitution of Thr 120 for Leu, which coincides with their nearly null activities. This result indicates that T120 and L120 play different roles. T120 is involved in the folding or stability of the protein; when it was substituted for Leu, less than 40% of the secondary structure and ~1% of the wild type enzyme activity remained. Therefore, this finding suggests that K^+^-dependent and K^+^-independent PKs have different folding or stability requirements.

### 2.5. Structural Features of the Open and Closed Conformations of WT-RMPK and of MtbPK

**Figure 6:**
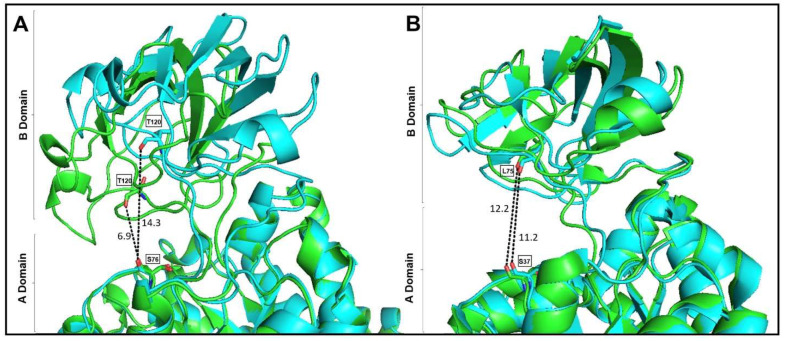
Ribbon representation of open and closed conformations of WT-RMPK (**A**) and of *Mtb*PK (**B**). According to the movement of the upper B domain over the central A domain of WT-RMPK, panels show the superposition of open/inactive (cyan) and closed/active (green) conformers. In both conformers, the distances between O of T120 to O S76 or O of L75 to S37 are shown. Panel A was constructed from the coordinates of PDB 1A49 subunit A (green) with its active site completely occupied (oxalate, Mg^2+^, -Mg-ATP, and K^+^) (closed conformation) and subunit B (cyan) with its active site partially occupied (oxalate, Mg^2+^and K^+^) (open conformation) [17]. Panel B was constructed from the coordinates of PDB 5WS9 subunit A (green) with its active site completely occupied (oxalate, Mg^2+^ Mg-ATP, and K^+^) and of PDB 5WRP subunit A (cyan) with its active site empty [18]. This figure was built using the program PYMOL v 2.3.1.

The skeletal muscle PK is constitutively active, and the first K^+^-dependent PK in which free ligand [26], partial ligand complex [27,28], and complete ligand complex [17] structural data states have been reported. Of the 97 crystal structures of PKs deposited in PDB [29], only three are K^+^-independent [18,30,31]. However, only the structural data of apo-, substrate-complex, effector-complex, and substrate-effector complex are available for *Mtb*PK [18]. It is well documented how ligands induce the closure of the active site cleft in WT-RMPK [17,20], but no information has been described in this respect in the K^+^-independent PKs. Figure 6A shows the overlapped structural conformations of WT-RMPK with partial ligand complex (cyan/open conformation) and complete ligand complex (green/closed conformation). In contrast, Figure 6B depicts the overlapped conformations of *Mtb*PK in the substrate-effector complex (green) and the apoenzyme (cyan). The RMPK holoenzyme structural data could not be compared with the crystal structure of the PK apoenzyme, as it is the cat´s muscle-derived [26]. Instead, the structural characteristics of the same crystal´s unit cell were compared. Figure 6A shows subunit 1 (green) with the active site completely occupied (K^+^, Mg^2+^, oxalate, and Mg-ATP) was overlapped with subunit 2 (cyan) that exhibits a partially occupied active site (K^+^, Mg^2+,^ and oxalate). B domain exhibited 0° and 41° angles of rotation relative to A domain in the former and latter subunits, indicating that the closure of the active site was either totally closed or totally open, respectively [17]. The distances between T120 and S76 in both conformations are shown. In WT-RMPK, T120 moves 7.4 Å in the closed conformation, whereas in *Mtb*PK, no movement of the B domain is induced by the substrate-effector complex. In this condition, L75 is even 1 Å farther than L75 in the apoenzyme (Figure 6B). 

This finding indicates that K^+^-dependent PKs follow a different mechanism to achieve the active conformation than the K^+^-independent PKs; T120 and L75 in the former and latter groups of PKs play different roles as indicated by the nearly null activity and differences in CD data described above. 

### 2.6. Molecular Dynamics Simulations of WT-RMPK, T120L Mutant and MtbPK in the Absence or Presence of Ligands

**Figure 7:**
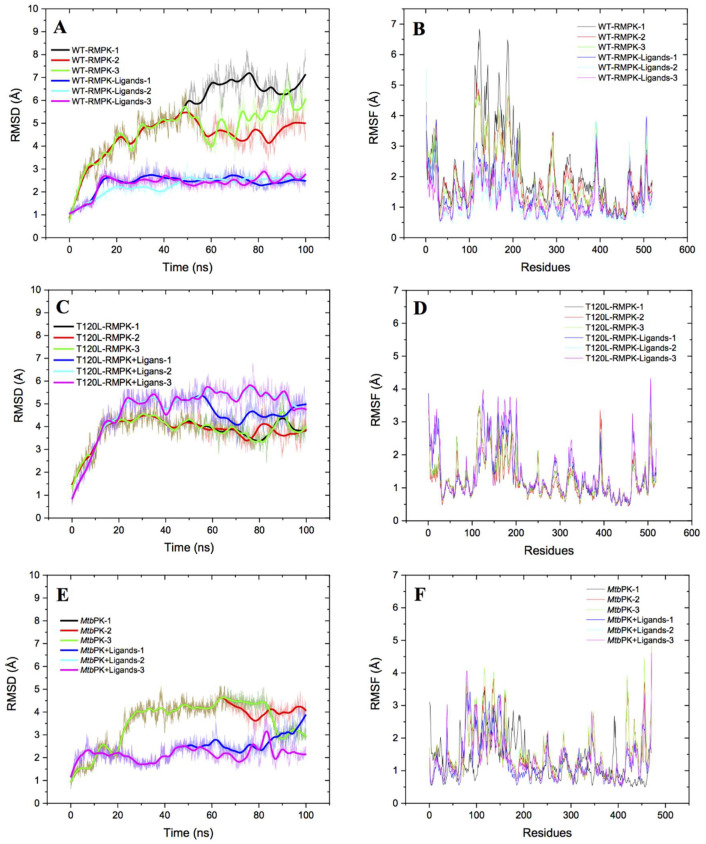
RMSD and RMSF from molecular trajectories of WT-RMPK (**A**,**B**), T120L mutant (**C**,**D**), and *Mtb*PK (**E**,**F**). Initial structures were in the presence of Mg^2+^, PEP, Mg-ADP and K^+^ for the K^+^-dependent WT-RMPK and T120L mutant and without K^+^ for the K^+^-independent *Mtb*PK. The presence or absence of ligands are indicated by different colors in (**A**,**C**,**E**), whereas red and blue lines indicated with and without ligands in (**B**,**D**,**F**). Triplicates of molecular dynamics were carried out and the analyzes were made with CPPTRAJ [32]. For the particular case of *Mtb*PK, runs 1 and 2 overlapped, either with or without ligands (**E**).

RMSD was used to evaluate global flexibility of structures. This analysis considers the average amount of movement of backbone atoms throughout the entire protein. RMSF estimates localized flexibility of individual amino acid residues throughout the overall structure. Three replicates of RMSD with and without ligands of WT-RMPK, T120L mutant, and *Mtb*PK are shown in Figure 7A,C,E, respectively. The flexibility of WT-RMPK without ligands increased ~5Å from 0 ns to 50 ns and showed fluctuations of 4 to 6 Å from 50 to 100 ns; whereas RMSD of the enzyme with ligands remained without change. In contrast, RMSD of T120L mutant with or without ligands kept constant through the time-lapse studied. RMSD of *Mtb*PK showed none and minimal changes with and without ligands, respectively, through the same period of time. On the other hand, RMSF in the presence (red) or in the absence (blue) of ligands of WT-RMPK, T120L mutant, and *Mtb*PK are shown in Figure 7 B,D,F, respectively. In WT-RMPK, a difference of ~4Å, was observed in the residues (116 to 223) corresponding to B domain (lid) with no ligands, compared to that with them. Contrary to observations in WT-PMPK, almost no differences were observed in the residues of the “lid” in T120L mutant and *Mtb*PK.

According to T120L mutant, although a native-like structure was simulated, it did not exhibit the flexibility of the lid characteristic of the K^+^-dependent PKs. Therefore, T120 is a critical residue for the closure of the lid in the catalysis of the K^+^-dependent PKs. In coincidence with Figure 6, the overall results of MD simulations indicate that K^+^-dependent and independent PKs follow different mechanisms to achieve the active conformation.

### 2.7. Structural Changes Induced by the Substitution of Thr for Leu 120 to the RMPK

As described before, RMPK was the first K^+^-dependent enzyme in which the structural data for the complete ligand complex was reported. It showed how the binding of the nucleotide brings the enzyme to a totally closed or active conformation [17]. Residues R119, K206 and D177, from the “lid”, move 6.8, 10.4 and 6.8 Å, respectively, from their position in the open conformation and establish contacts to the nucleotide in the closed conformation. K206 NZ moves 10.4 Å to a position between the 2′- and 3′-hydroxyls of the ribose moiety; the guanidinium moiety of R119 moves 6.8 Å to H-bonding distance of the β-phosphate of the nucleotide; and the carboxylate of D177 travels 6.8 Å to H-bonding contact with a water of hydration on the Mg^2+^ of Mg-ATP. The adenine ring fits into a pocket bounded on one side by His 77 [17].

**Figure 8:**
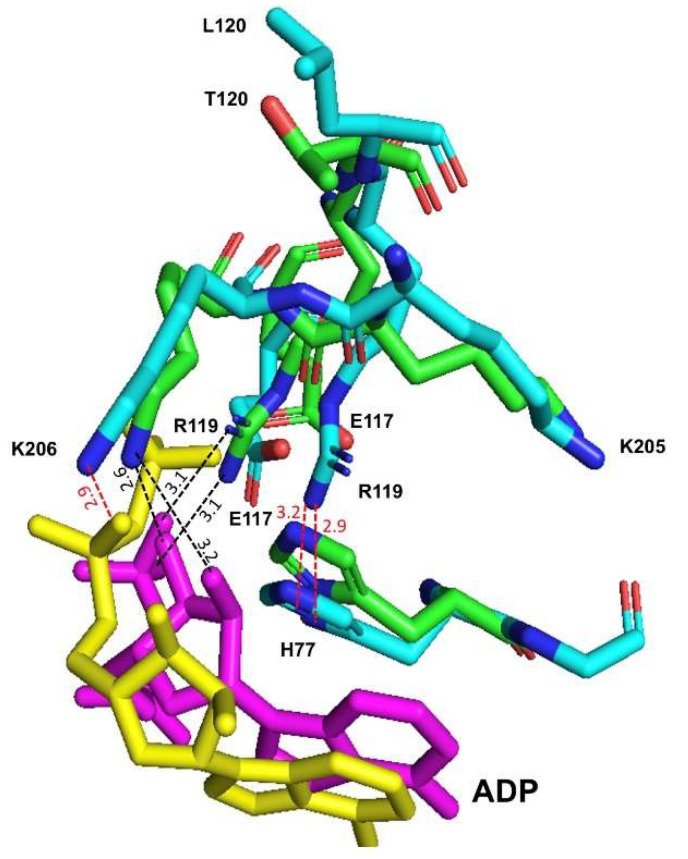
Comparison of the interactions of ADP (magenta and yellow) in the active site of the WT-RMPK (green) and T120L mutant (cyan). In the WT-RMPK, NH_2_ of R119 is at H-bond distance (3.13 and 3.11 Å) of O3B and O1B of ADP, respectively. In contrast, in the T120L mutant, NH_2_ of R119 is at 2.92 and 3.16 Å of ND1 and CE1 of H77, respectively. In WT-RMPK, NZ of K206 is at H-bond distance (2.57 and 3.19 Å) of O3´ and O2´of ribose, respectively; whereas in T120L mutant, NZ of K206 is at H-bond distance (2.96 Å) of O1A of ADP. This figure was prepared using the molecular dynamic models of both enzymes in the initial structures described in Figure 7 from PDB 149A.

Figure 8 compares the superposition of the molecular dynamic models of WT-RMPK and T120L mutant in the initial structures with Mg^2+^, PEP, Mg-ADP and K^+^ to understand the differences caused by this single mutation. In the T120L mutant, a structural distortion of the guanidium moiety of R119 was observed. It moved to the imidazole moiety of His77 instead of the β-phosphate of ADP. NZ of K206 moved to position of H-bonding distance of the ADP α-phosphate instead of moving between the 2′- and 3′-hydroxyls of the ribose moiety. The missing and new interactions between nucleotide and residues from the “lid” could explain the almost null activity of the T120L mutant.

### 2.8. Different Main Chain Orientations of Residue Lys117 in the E117K Mutant and the Corresponding Lys72 in the Apo and Holoenzyme of MtbPK

The structure of the E117K mutant shows a tetramer in the asymmetric unit. Subunits A and B are the most complete ones with 519 residues out of 530, missing only the first 12 residues at the N-terminus. Due to poor density, subunit C lacks 121 amino acids, missing 11 residues at the N-terminus and from 121 to 203. Likewise, subunit D lacks 11 residues at the N-terminus, and it is missing the amino acids 118 to 215.

A superposition of subunits A and B shows almost identical structures with RMSD of 0.25 Å. However, amino acids 111 to 220 are the most different in this alignment and do not superimpose perfectly. Thus, a different conformation of the NZ atom in K117 is observed in both subunits. The NZ atom in subunit B is within H-bond distance with a water molecule, which coordinates to the Mg^2+^ ion near the oxalate ligand. Such an interaction does not occur in subunit A, which has much fewer water molecules in the vicinity of the oxalate.

In contrast, K117 in subunit C exhibits similar conformation to the one observed in subunit B, but shifted by about 0.5 Å. Consequently, the “lid” domain was not observed, likely due to high flexibility and a lack of complete electron density. Finally, residues K117-215 in subunit D could not be interpreted in the electron density. These couple motions of the “lid” domains could define functional transitions.

**Figure 9:**
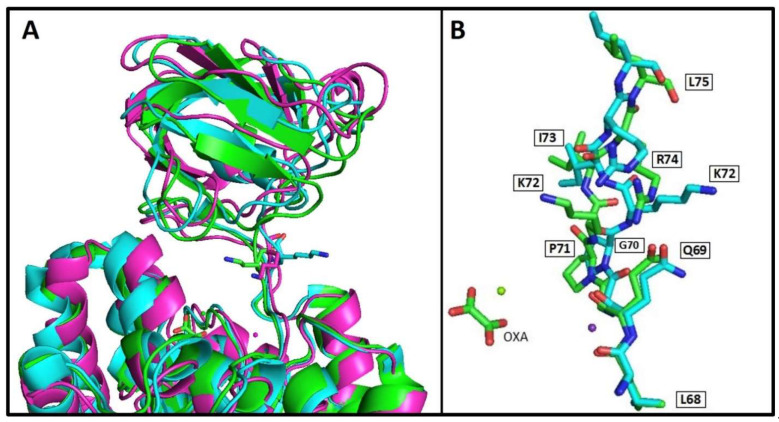
Comparison between residues K117 in E117K mutant (magenta), K72 in the apo (cyan), and holoenzyme (green) in *Mtb*PK (**A**). Close up of residues L68 to L75 of *Mtb*PK forming part of the short loop that connects A domain to B domain in the apo (cyan) and holoenzyme (green) on *Mtb*PK (**B**). In (**A**), the superposition of the E117K mutant coordinates (PDB 7R6Y subunit A; magenta), the holo-*Mtb*PK (PDB 5WS9 subunit A; green), and the apo-*Mtb*PK (PDB 5WRP subunit A; cyan) (18) is shown. Residue K72 in *Mtb*PK corresponds to residue 117 in E117K mutant. The holoenzyme (green) contained oxalate, Mg^2+,^ Mg-ATP, and K^+^; however, the nucleotide was omitted. The oxalate and Mg^2+^ present in the RMPK mutant are not shown. In (**B**), the figure shows that apo (cyan) or holoenzyme residues overlap or have the same orientation in the loop except for K72. This figure was prepared using PYMOL v 2.3.1.

When the *Mtb*PK structure was compared, it was evident that K72 (corresponding to the residue 117 of the RMPK mutant) is positioned towards the active site in the holo- and flips out in the apoenzyme. The partially occupied mutant E117K mutant is also facing toward the active site (Figure 9A). The distances between NZ of K72 (or K117) to O1 of the oxalate are 7.4 and 7.2 Å, respectively.

On the other hand, it is relevant to mention that T113 to T120 are localized in a loop bending region that participates in the closure or acquisition of the active catalytic conformation of K^+^-dependent holo-WT-RMPK (Figure 6A), whereas no bending is observed in this loop region in K^+^-independent holo-*Mtb*PK (Figure 6B). In the latter, loop residues, L68-L75, exhibit the same orientation in the apo- or holoenzyme, except for K72 that flips into or out of the active site, respectively (Figure 9B). This finding indicates that a completely different mechanism is observed in the K^+^-independent *Mtb*PK, where K72 switches from the inactive (apo) to the active (holo) conformation.

### 2.9. Different Interaction of MgATP to Oxalate, Mg^2+^ and K^+^ in the Active Sites of WT-RMPK and MtbPK

**Figure 10:**
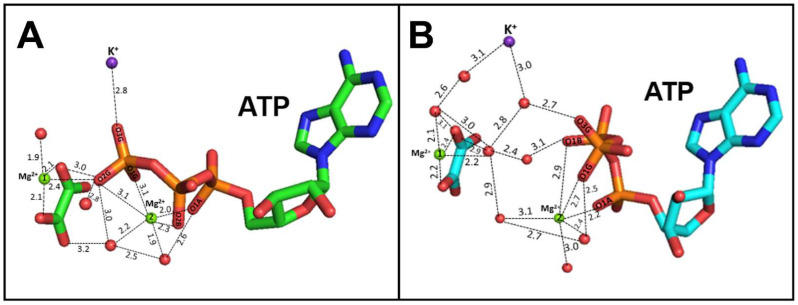
Differential coordination of Mg-ATP in the active site of a K^+^-dependent PK (WT-RMPK) (**A**) and in a K^+^-independent PK (*Mtb*PK) (**B**). Panel A and Panel B show the coordination of oxalate, Mg^2+^, Mg-ATP, and K^+^ in the active sites of RMPK (PDB 1A49 subunit A; green) and of *Mtb*PK (PDB 5WS9 subunit A; cyan), respectively. No residues of the enzymes are shown.

In WT-RMPK, the γ-phosphate of Mg-ATP exhibits direct interaction with oxalate, Mg^2+^, and K^+^ (Figure 10A), whereas in *Mtb*PK these interactions occur through water molecules (Figure 10B). The latter observation suggests that water molecules may have a relevant role in the catalysis of the K^+^-independent enzymes.

### 2.10. Active Sites of the Holo and Apo MtbPK

**Figure 11:**
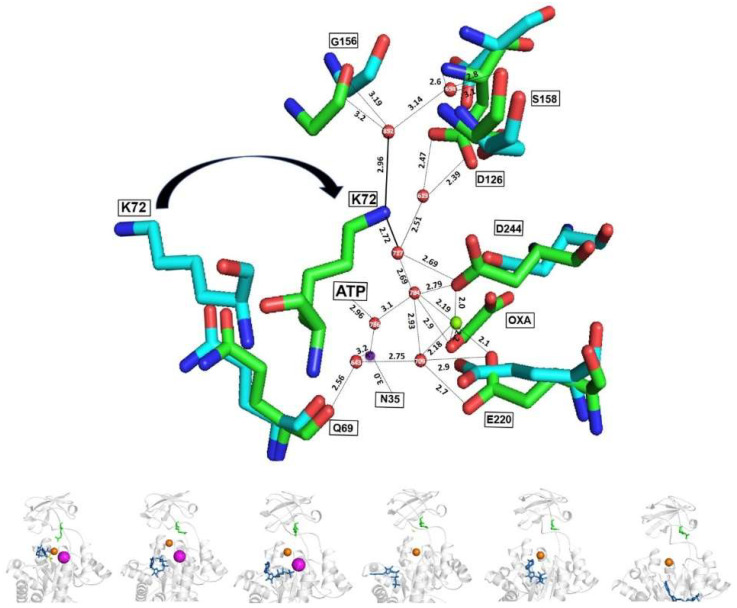
Ribbon representation of the active sites of *Mtb*PK in the holo (green) and the apoenzyme (cyan); and MD. Superposition of the coordinates of the holo- (PDB 5WS9 subunit A; green) to the apoenzyme (PDB 5WRP subunit A; cyan) are shown. As observed in Figure 9, K72 flips out or into the active site in the apo- and holoenzyme, respectively. ε-amino group of K72 interacts with Mg^2+^, Oxalate, Mg-ATP, K^+^, residues of A domain and of the lid (B domain) via a water network. Numbering is according to *Mtb*PK. The bottom part shows the structural models of the MD of *Mtb*PK every 20 ns, observing the same displacement of the K72 (green sticks). Ligands are represented as follows: ATP (blue sticks) Oxalate (yellow sticks), Mg^2+^ (orange spheres) and K^+^ (magenta spheres).

As shown in Figure 11, K72 in the apoenzyme flips out of the active site. In contrast, in the holoenzyme, K72 faces toward the active site, interacting with a network of water molecules connecting the lid (domain B), substrates (Mg^2+^, oxalate, Mg-ATP, and K^+^), and A domain residues. Therefore, K72 is a key residue in the water network formation. A similar water network is observed in the crystal structure of K^+^-independent PK of *Pseudomonas aeruginosa* [31]. Consequently, this data confirmed that the acquisition of the active conformation of the K^+^-independent PKs is via K72 switching from the inactive to the active conformation.

## 3. Conclusions

In summary, the aim of this work was to understand the individual and combined role of changing specific residues in WT-RMPK to those observed in K^+^-independent PKs. K^+^-independent activity increased with residues L113 and Q114 to K117, with activity in the absence of K^+^ reaching half WT-RMPK activity with K^+^. In contrast, the single mutant T120L or the addition of this mutation to other mutants leads to a nearly inactive enzyme. Single mutants, T113L and K114Q, were K^+^-dependent with kinetic constants similar to WT-RMPK, except that ion selectivity for T113L was modified as described elsewhere [21]. Furthermore, residues in positions 117 and 120 appear decisive in the differences between the K^+^-dependent and -independent PKs. When Leu substituted T120, the enzyme exhibited different structural features with nearly null activity and did not close the lid to acquire its active conformation. Although simulations indicated it to be folded entirely, no movement of the residues of the “lid” was observed by MD. We conclude that T120 is involved in folding or stabilizing the protein and closing the lid of the active site in the K^+^-dependent PKs. When Lys was substituted for E117, the enzyme became K^+^-independent and acquired its active conformation by a different mechanism. The holoenzyme with E117 acquires its active conformation by closing the B domain over A domain, whereas in the K^+^-independent *Mtb*PK, K72 (or K117) switches between the active and inactive conformations. In the apoenzyme, K72 flips out of the active site and, in the holoenzyme, K72 faces into the active site, connecting the substrates, residues of B domain, and A domain via water molecules. Finally, the K^+^ binding site is highly conserved in both branches, K^+^-dependent and K^+^-independent, of the phylogenetic tree of the PK family. In most K^+^-independent PKs, three of the four coordination residues of K^+^ are preserved. The role of K^+^ is not catalytically essential like in K^+^-dependent PKs, but may exert a weak ionic strength activation, as seen in the K^+^-independent mutants studied in this work.

## 4. Experimental Procedures

### 4.1. Cloning and Expression of RMPK Mutants

In all cases (Table 3), Fw and Rv mutagenic primers and external primers were used for PCR reaction. Mutagenesis was performed using the following PCR conditions: 94 °C for 5 min, 25 cycles for 30 s at 94 °C, 30 s at 62 °C, 1.5 min at 72 °C and 5 min at 72 °C. The PCR products were cloned in the pJET 1.2 vector, and mutagenesis was confirmed by automated DNA sequencing of the complete genes. The pJET 1.2 vector containing the verified sequence for each mutant was digested with EcoRI and XbaI and subcloned into the pTRC99A plasmid. All constructs with the desired mutations were transformed into competent PB25 cells for protein expression.

### 4.2. Cell Growth and Purification of RMPK Mutants

LB medium containing 100 μg/mL ampicillin, 50 μg/mL kanamycin, and 30 μg/mL chloramphenicol was inoculated either with PB25-E117K, or the other mutants. Expression was induced with 1 mM isopropyl 1-thio-β-D-galactopyranoside and 20 g/liter of lactose at an OD_600_ of about 0.8. The enzyme was purified as in [20] with some modifications. Cells were treated with lysozyme and an osmotic shock. DNAse and a protease inhibitor mixture were added to the spheroplasts, which were then broken by sonication. The suspension was centrifuged for 20 min at 20,000× *g*. The supernatant was precipitated with ammonium sulfate at 37%, and the resultant supernatant was collected. A second precipitation with ammonium sulfate at 65% saturation was prepared. The pellet was collected, suspended, and desalted by dialysis. The final steps involved ion exchange chromatography in DEAE and carboxymethyl-Sepharose columns previously equilibrated with HEPES pH 6.4 to 6.6 for all mutants except for the mutants containing the substitution of Thr 120 by Leu (T120L, K114Q/E117K/T120L and T113L/K114Q/E117K/T120L mutants). These mutants were poorly expressed, and about half of the proteins were in inclusion bodies. To obtain more solubilized enzymes, 500 mM NaCl was added to the buffer before lysis. The columns were equilibrated at pH 6.9, and 10% glycerol was included in all purification steps. Unlike the ~15 mg/ 4 L LB broth media recovered for other mutants, those with the T120L mutation recovered only ~2 mg/4 L. The mutants were about 95% pure, as indicated by SDS-PAGE (not shown). Purified K114Q, E117K, T113L/E117K, and T113L/K114Q/E117K mutants were stored as ammonium sulfate suspensions at 4 °C. Ammonium sulfate-free T120L exhibited no activity. Therefore, further purifications of T120L, K114Q/E117K/T120L and T113L/K114Q/E117K/T120L were stored in 20% glycerol at −20 °C in aliquots of 50 μl. However, K114Q/E117K/T120L was completely inactive and T120L and T113L/K114Q/E117K/T120L were nearly inactive. K114Q/E117K [15] and T113L [21] were not included in this study because they were studied previously.

### 4.3. Assays of Pyruvate Kinase Activity

Ammonium sulfate suspensions of WT-RMPK and hog muscle lactate dehydrogenase (LDH) were obtained as ammonium sulfate suspensions from SIGMA. Ammonium sulfate-free enzymes (WT-RMPK, LDH, K114Q, E117K, T113L/E117K, and T113L/K114Q/E117K) were obtained as described in [33]. Contaminating NH_4_^+^, Na^+^, and K^+^ in reaction mixtures were below the detection limit (10 μM) as indicated in [33]. Mutants containing the substitution of Thr by Leu in position 120 (T120L, K114Q/E117K/T120L and T113L/K114Q/E117K/T120L mutants) exhibited none or hardly any activities. The formation of pyruvate was measured at 25 °C in a coupled system with LDH and NADH [34]. The specific activity was not increased by the inclusion of 5-fold higher concentrations of LDH.

The reaction mixture contained 25 mM HEPES-(CH_3_)_4_NOH, pH 7.4, and the concentrations of monovalent cations (Li^+^, Na^+^, K^+^, NH_4_^+^, Rb^+^, and Cs^+^), Mg^2+^, phosphoenolypyruvate (PEP), and ADP indicated in each figure legend. The Mg-ADP complexes and free Mg^2+^ concentrations were calculated using the software CHELATOR [35]. The ionized PEP concentrations were calculated considering a pK value of 6.3 [36]. With the exception of Figure 2 and Figure 3, (CH_3_)_4_N^+^ was used to maintain at 250 mM constant ionic strength. The experiments were carried out at 25 °C, and the reaction was initiated with the addition of either RMPK or any mutant.

Protein concentrations of RMPK and of mutants were determined by measuring the absorbance at 280 nm using the extinction coefficients of 0.54 mL mg^−1^ cm^−1^ [37].

### 4.4. Circular Dichroism Experiments

CD measurements in the far-ultraviolet (UV) region were carried out on a Jasco J-715 spectropolarimeter. A 0.1-cm quartz cell was used and the experiments were conducted at 25 °C. The concentration of WT-RMPK and of RMPK mutants: K114Q, E117K, T120L, T113L/E117K, T113L/K114Q/E117K was K114Q/E117K/T120L, and T113L/K114Q/E117K/T120L was 100 μg/mL. Spectral scans were run from 190 to 260 nm at intervals of 1 nm, scan rates of 20 nm/min, and a time constant of 16 s. In each case, bandwidth was 1 nm, and three scans were accumulated. The spectra of blanks were subtracted from those that contained the protein. CD is expressed as θ_MRW_ (mean residual weight ellipticity).

### 4.5. Molecular Dynamics Simulation

#### 4.5.1. Preparation of Initial Coordinate Files

The coordinates corresponding to the structure of RMPK and *Mtb*PK were obtained from the Protein Data Bank (PDB, http://www.rcsb.org) (accessed on 13 January 2022). The structure used was 1A49.pdb refined at 2.1 Å [17] for RMPK and PDB entry 5ws9 for *Mtb*PK (refined at 1.9 Å). The structures had the hydrogens removed before the initial preparation of the molecular dynamics (MD). The T120L mutant was constructed from the same structure (PDB entry 1A49.pdb) using the PyMOL program [38], and subsequently refined with Rosetta relax 3.4 [39]. The structures of the ligands ADP, ATP, oxalate, and PEP were obtained from the PDB co-crystallized structure when available or constructed using *Spartan’16* Parallel Suite for Linux software. All structures were minimized using Gaussian 09, revision A.02 at DTP B3LYP/3-21G level of theory. Partial charges and force fields of the ligands were generated using the antechamber program in AmberTools19.

#### 4.5.2. Molecular Dynamics Simulation

All the structural complexes were verified, cleaned, and ordered with the *pdb4amber* scrip before starting the preparation in order to generate suitable topologies from the LEaP module of AmberTools19 [40,41,42]. Each structure and complex were subjected to the following protocol: hydrogen and other missing atoms were added using the LEaP module with the leaprc.protein.ff19SB parameter set, Cl^−^ counterions were added to neutralize the system; the complexes were then solvated in an octahedral box of explicit TIP3P model water molecules localizing the box limits at 12 Å from the protein surface. MD simulations were performed at 1 atm and 298.15 K, maintained with the Berendsen barostat and thermostat, using periodic boundary conditions and particle mesh Ewald sums (grid spacing of 1 Å) for treating long-range electrostatic interactions with a 10 Å cutoff for computing direct interactions. The SHAKE algorithm was used to satisfy bond constraints, allowing the employment of a two fs time step for the integration of Newton’s equations as recommended in the Amber package [41,42]. Amber leaprc.protein.ff19SB force field [43] parameters were used for all residues. All calculations were made using graphics processing units (GPU) accelerated MD engine in AMBER (*pmemd.cuda*), a program package that runs entirely on CUDA-enabled GPUs [44]. The protocol consisted of performing a minimization of the initial structure, followed by 2500 heating steps (50 ps) and depressurization (50 ps) at 298.15 K and 1.0 atm pressure, respectively. Finally, the system is equilibrated with 250,000 steps (500 ps) before starting the production of MD. The production of the MD consisted of 100 ns (50,000,000 steps) for each complex. Frames were saved at ten ps intervals for subsequent analysis. Three replicates of the molecular dynamics simulations were carried out. Each of them starting from the minimization step. All data from molecular dynamics studies are available at the following link http://bq.unam.mx/ijms-23-01347/ (accessed on 13 January 2022).

#### 4.5.3. Trajectory Analysis

All analyses were done using CPPTRAJ [45] part of AmberTools19 utilities and OriginPro 9.1. The calculations of RMSD and Root Mean Square Fluctuations (RMSF) were made, considering the C, CA, and N; for the distances, only the CA was used. The charts were built with Origin 9.0, and the trends were adjusted with the function processing smooth (method Lowess). VMD and PyMOL were used to visualize and create the images from the MD.

All calculations were made using a system HP Cluster Platform 3000 SL supercomputer “MIZTLI” with a processing capacity of 118 TFlop/s. It has 5312 Intel E5-2670 processing cores, 16 NVIDIA m2090 cards, a total RAM of 15,000 Gbytes, and a mass storage system of 750 Terabytes (http://www.super.unam.mx/) (accessed on 13 January 2022).

### 4.6. Crystallization of the Mutant E117K

The pure E117K mutant was dialyzed into a buffer containing 50 mM Tris pH 7.0. The enzyme was concentrated in the same buffer, and 1.0 mM ATP, 1.0 mM MgCl_2_, and 0.2 mM oxalate were added to the protein solution, which ended with a final concentration of 7 mg/mL. The vapor diffusion method in a hanging drop mode was used to set up the crystallization drops, using 24 well plates. Wells contained 500 μL of crystallization buffer, and drops were 4.0 μL with a 1:1 ratio of enzyme to crystallization buffer. After the first set of crystallization experiments, the best-looking crystals appeared in condition No. 46 of the Hampton Research Crystal Screen I (Journey Aliso Viejo, CA, USA). This solution contains 0.2 M calcium acetate, 0.1 M sodium cacodylate pH 6.5, and 18% w/v Polyethylene Glycol 8000. However, diffracting-quality crystals grew from drops containing 0.2 M magnesium acetate instead of calcium acetate. For this experiment, the well´s crystallization solution was covered with a 1:1 mixture of paraffin and silicone oils from Hampton Research (Journey, Aliso Viejo, CA, USA).

### 4.7. Data Collection and Reduction

X-ray diffraction data for the best crystals were collected on beamline X6A at the National Synchrotron Light Source (Upton, NY, USA), at 100 K using 25% glycerol as cryoprotectant. A Quantum 210 CCD detector was used to collect 180 images with an oscillation range of 1.0° and 230 mm from the detector. Frames were integrated using XDS [46], with an initial space group P222, which was confirmed as P212121 using pointless in CCP4 [47]. Data were scaled with SCALA from CCP4 [47], and the mtz file was analyzed using Xtriage from Phenix [48], which suggested no problems with the data or space group selected. The Matthew’s coefficient of 2.2 and 0.45 fraction of solvent suggested four copies of the E117K pyruvate kinase mutant in the asymmetric unit. Data collection statistics are shown in Table 4.

### 4.8. Molecular Replacement and Model Refinement

Initial phases for the orthorhombic P212121 were determined by molecular replacement using chain A of the pyruvate kinase´s atomic coordinates from rabbit muscle (PDB 1AQF) with the program Phaser from Phenix [49]. A tetramer of the E117K mutant was obtained as the solution. The initial tetrameric model was refined using rigid-body refinement, and non-crystallographic (NCS) restraints were then applied. The resulting model underwent iterative rounds of refinement with Phenix.refine and manual rebuilding with Coot [50,51]. After the initial stages of refinement, oxalate, acetate, solvent, and ions were included, and the CCP4 cif files for all molecules except water were used during refinement. Refinement was finished when the best possible Rfree and Rwork statistics were obtained, and further adjustments no longer improved the model. Validation of the Mg^2+^ metal ions was done using the CheckMyMetal server [52].

## Figures and Tables

**Table 1:** Kinetics of the activation of WT-RMPK and K114Q mutant by monovalent cations at saturating concentrations of PEP^3−^, Mg-ADP complex, and Mg^2+^_free_.The data from Figure 3A were fitted to the Michaelis–Menten equation (*v = V_max_* *S/K_m_* + *S*) and to the Hill equation (*v = V_max_ S^n^/K*_0.5_*^n^*+ *S^n^*) (Origin version 7.0). The mean and standard deviation from three experiments are shown. *k_cat_*/*^a^K* values are expressed in log form.

M^+.Ionic radius^ (pm)	WT-RMPK				K114Q			
	*k_cat_* (s^−1^)	*K* (mM)	n	log E (s^−1^ M^−1^)	*k_cat_* (s^−1^)	*K* (mM)	n	log E (s^−1^ M^−1^)
Li^+^_69_	16 ± 3	53 ± 25	___	0.47	N.D.	N.D.	N.D.	N.D.
Na^+^_102_	138 ± 8	48 ± 4	1.7 ± 0.1	3.46	N.D.	N.D.	N.D.	N.D.
K^+^_138_	857 ± 16	5.8 ± 0.2	1.5 ± 0.1	5.17	970 ± 24	11 ± 0.6	1.4 ± 0.1	4.95
NH_4_^+^_147_	596 ± 24	2.5 ± 0.2	1.8 ± 0.2	5.38	742 ± 25	3.5 ± 0.2	1.6 ± 0.1	5.33
Rb^+^_148_	462 ± 20	5.4 ± 0.4	1.4 ± 0.2	4.93	691 ± 24	7.0 ± 0.5	1.5 ± 0.1	4.99
Cs^+^_170_	198 ± 12	20 ± 3	1.5 ± 0.1	3.99	223 ± 13	15 ± 1.8	1.4 ± 0.2	4.17

^a^*K* represents the *K*_0.5_ and *K_m_* for the data fitted to the Hill and Michaelis–Menten equations, respectively.

**Table 2:** Kinetic constants for PEP^3−^, MgADP complex and Mg^2+^_free_ of WT-RMPK, K114Q-RMPK, E117K-RMPK, T113L/E117K-RMPK and T113L/K114Q/E117K-RMPK. The experiments for PEP^3−^ were carried out in the presence of saturating MgADP complex and saturating Mg^2+^_free_, whereas those for MgADP and Mg^2+^_free_ were performed in the presence of saturating concentrations of the other substrates. The experimental conditions were as in Figure 2 except for the absence of monovalent cations for pyruvate kinases mutants with K117 (K^+^-independent enzymes) and the inclusion of (CH_3_)_4_NCl to maintain ionic strength at 250 mM. Kinetic data (not shown) were fitted to the Hill equation (*v = V_max_ S^n^/K_0.5_^n^* + *S^n^*) and to the Michaelis–Menten equation (*v = V_max_* *S/K_m_* + *S*) (Origin version 7.0). The mean and standard deviation from three experiments are shown. *k_cat_*/*^a^K* values are expressed in log form. Data of ^a^T113L and ^b^T113L were taken from Table S1 and Table S2 of Supplementary material, respectively, [21].

RMPK & RMPK Mutants	With E117				RMPK Mutants	With K117			
	*k_cat_* (s^−1^)	*K* (mM)	*n*	Log *k_cat/_K* (s^−1^ M^−1^)		*k_cat_* (s^−1^)	*K* (mM)	*n*	Log *k_cat/_K* (s^−1^ M^−1^)
	PEP					PEP			
WT	703 ± 28	0.063 ± 0.008	__	7.05	E117K	249 ± 16	0.063 ± 0.00	__	6.60
^a^T113L	725 ± 49	0.108 ± 0.018	__	6.83	T113L/E117K	280 ± 8	0.048 ± 0.002	__	6.77
K114Q	805 ± 26	0.15 ± 0.014	__	6.74	T113L/K114Q/E117K	363 ± 4	0.067 ± 0.00	1.82 ± 0.17	6.73
**MgADP**						**MgADP**			
WT	691 ± 16	0.23 ± 0.010	__	6.48	E117K	261 ± 12	0.360 ± 0.035	1.23 ± 0.09	5.86
^b^T113L	831 ± 120	1.07 ± 0.26	__	5.89	T113L/E117K	363 ± 28	0.100 ± 0.030	__	6.56
K14Q	690 ± 19	0.54 ± 0.034	__	6.10	T113L/K114Q/E117K	419 ± 8	0.640 ± 0.030	1.82 ± 0.17	5.82
**Mg^2+^_free_**						**Mg^2+^_free_**			
WT	865 ± 12	0.17 ± 0.010	1.46 ± 0.1	6.71	E117K	284 ± 4	4.9 ± 0.3	__	4.76
K114Q	N.D.	N.D.	N.D.	N.D.	T113L/E117K	458 ± 75	1.6 ± 0.5	1.39 ± 0.3	5.46
T113L	N.D.	N.D.	N.D.	N.D.	T113L/K114Q/E117K	371 ± 16	8.7 ± 0.7	1.44 ± 0.1	4.63

*K* represents the *K*_0.5_ and *K_m_* for the data fitted to the Hill and Michaelis–Menten equations, respectively.

**Table 3:** List of DNA templates and primer sequences used in PCR.

Name	Template DNA	Primer Sequence 5′→3′
K114Q	WT-RMPK	FW_CTGGACACTCAGGGACCCGAG Rv_CTCGGGTCCCTGAGTGTCCAG
T120L	WT-RMPK	Fw_ATCCGGCTAGGCCTCATCAAGGGC Rv_GCCCTTGATGAGGCCTAGCCGGAT
T113L/E117K	E117K mutant ^1^	Fw_CTGGACCTTAAGGGACCCAAGATCCGGACG Rv_AGTCCGGATCTTGGGTCCCTTAAGGTCCAG
T113L/K114Q/E117K	E117K mutant ^1^	Fw_CTCTGGACCTGCAGGGACCC Rv_GGGTCCCTGCAGGTCCAGAG
K114Q/E117K/T120L	K114Q mutant	Fw_GGACCCAAGATCCGGCTAGGCCTCATCAAGGGC Rv_GCCCTTGATGAGGCCTAGCCGGATCTTGGGTCC
T113L/K114K/E117K/ T120L	T113L/K114Q/E117K mutant	
External (pTRC99A)		Fw_TAATCATCCGGCTCGTATAATGTG Rv_GGCTGAAAATCTTCTCTCATCCGC

^1^ Reference [20].

**Table 4:** Data collection and refinement statistics.

**Data Collection**	
Wavelength [Å]	0.98
Space group	P212121
Unit cell dimensions a, b, c [Å]	108.34, 121.83, 161.37
Resolution range [Å] ^a^	48.18–2.25 (2.37–2.25)
R_merge_ (%) ^b^	0.093 (0.57)
I/σI	17.5 (3.0)
Observations Total Unique	733102 (89732) 101836 (14698)
Completeness	99.9 (100.0)
Redundancy	7.2 (6.1)
CC1/2	0.99 (0.81)
**Refinement Statistics**	
Rwork/Rfree (%) ^c^	0.20/0.25
No. of atoms:	15009
Protein	14307
Ligands	60
Metal Ions	4
Solvent	638
B value (Å^2^)	34.0
RMSD bond lengths (Å)	0.006
RMSD bond angles (°)	0.898
Ramachandran favored (%)	98.73
Ramachandran allowed (%)	1.11
Ramachandran outliers (%)	0.16
PDB code	7R6Y

^a^ Statistics for the highest resolution shell is shown in parenthesis. ^b^ R_merge_ = Σ_hkl_Σ_i_ |I_i_(hkl)–<I(hkl)> | /Σ_hkl_Σ_i_ I_i_(hkl) ^c^ R_free_ was calculated from a subset of reflections (4.9%), which were not used during refinement.

## Data Availability

The data that support the findings of this study are available from the corresponding author upon reasonable request.

## Abbreviations

Pyruvate kinase	PK
Wild type rabbit muscle pyruvate kinase	WT-RMPK
Choline	(CH_3_)_3_N^+^-CH_2_-CH_2_-OH
Circular dichroism	CD
Molecular dynamics	MD
*Mycobacterium tuberculosis* pyruvate kinase	*Mtb*PK
N-(2-Hydroxyethyl)piperazine-N′-(2-ethanesulfonic acid)	HEPES
phosphoenolpyruvate	PEP
Luria Bertani	LB
hog muscle lactate dehydrogenase	LDH

## References

[1] Kachmar J.F., Boyer P.D. (1953). Kinetic analysis of enzyme reactions: II. The potassium activation and calcium inhibition of pyruvic phosphoferase. J. Biol. Chem..

[2] Ramírez-Silva L., Ferreira S.T., Nowak T., Tuena de Gómez-Puyou M., Gómez-Puyou A. (2001). Dimethyl sulfoxide promotes the K^+^-independent activity of pyruvate kinase and the acquisition of the active catalytic conformation. Eur. J. Biochem..

[3] Kayne F.J. (1971). Thallium (I) activation of pyruvate kinase. Arch. Biochem. Biophys..

[4] Nowak T. (1976). Conformational changes required for pyruvate kinase activity as modulated by monovalent cations. J. Biol. Chem..

[5] Benziman M. (1969). Factors affecting the activity of pyruvate kinase of *Acetobacter xylinum*. Biochem. J..

[6] Liao C.L., Atkinson D.E. (1971). Regulation at the phosphoenolpyruvate branchpoint in *Azotobacter vinelandii*: Pyruvate kinase. J. Bacteriol..

[7] Smart J.B., Pritchard G.G. (1979). Regulation of pyruvate kinase from *Propionibacterium shermanii*. Arch. Microbiol..

[8] Kapoor R., Venkitasubramanian T.A. (1981). Glucose 6-phosphate activation of pyruvate kinase from *Mycobacterium smegmatis*. Biochem. J..

[9] Waygood E.B., Rayman M.K., Sanwal B.D. (1975). The control of pyruvate kinase of Escherichia coli. II. Effectors and regulatory properties of the enzyme activated by ribose 5-phosphate. Can. J. Biochem..

[10] Busto F., Del Valle P., Soler J. (1988). Some kinetic properties of pyruvate kinase from *Phycomyces blakesleeanus*. Biochem. Cell Biol..

[11] Jetten M.S.M., Gubler M.E., Lee S.H., Sinskey A.J. (1994). Structural and functional analysis of pyruvate kinase from *Corynebacterium glutamicum*. Appl. Environ. Microbiol..

[12] Steiner P., Fussenegger M., Bailey J.E., Sauer U. (1988). Cloning and expression of the *Zymomonas mobilis* pyruvate kinase gene in *Escherichia coli*. Gene.

[13] Schramm A., Siebers B., Tjaden B., Brinkmann H., Hensel R. (2000). Pyruvate kinase of the hyperthermophilic Crenarchaeote *Thermoproteus tenax*: Physiological role and phylogenetic aspects. J. Bacteriol..

[14] Johnsen U., Hansen T., Schönheit P. (2003). Comparative analysis of pyruvate kinases from the hyperthermophilic archaea *Archaeoglobus fulgidus*, *Aeropyrum pernix*, and *Pyrobaculum aerophilum*. J. Biol. Chem..

[15] Laughlin L.T., Reed G. (1997). The monovalent cation requirement of rabbit muscle pyruvate kinase is eliminated by substitution of lysine for glutamate 117. Arch. Biochem. Biophys..

[16] Oria-Hernández J., Riveros-Rosas H., Ramírez-Silva L. (2006). Dichotomic phylogenetic tree of the pyruvate kinase family. K^+^-dependent and –independent enzymes. J. Biol. Chem..

[17] Larsen T.M., Benning M.M., Rayment I., Reed G.H. (1998). Structure of the bis(Mg^2+^)-ATP-oxalate complex of the rabbit muscle pyruvate kinase at 2.1 Å resolution: ATP binding over a barrel. Biochemistry.

[18] Zhong W., Cui L., Goh B.C., Cai O., Ho P., Chionh Y.H., Yuan M., El Sahili A., Fothergill-Gilmore L., Walkinshaw M.D. (2017). Allosteric pyruvate kinase-based “logic gate” synergistically senses energy and sugar levels in *Mycobacterium tuberculosis*. Nat. Commun..

[19] Suelter C.H., Sigel H. (1974). Monovalent cations in enzyme-catalyzed reactions. Metal Ions in Biological Systems.

[20] Oria-Hernández J., Cabrera N., Pérez-Montfort R., Ramírez-Silva L. (2005). Pyruvate kinase revisited. The activating efffect of K^+^. J. Biol. Chem..

[21] Ramírez-Silva L., Guerrero-Mendiola C., Cabrera N. (2014). The importance of polarity in the evolution of the K^+^-binding site of pyruvate kinase. Int. J. Mol. Sci..

[22] Di Cera E. (2006). A structural perspective on enzymes activated by monovalent cations. J. Biol. Chem..

[23] Page M.J., Di Cera E. (2006). Role of Na^+^ and K^+^ in enzyme function. Physiol. Rev..

[24] Guerrero-Mendiola C., García-Trejo J.J., Encalada R., Saavedra E., Ramírez-Silva L. (2017). The contribution of two isozymes to the pyruvate kinase activity of *Vibrio cholera*: One K^+^-dependent constitutively active and another K^+^-independent with essential allosteric activation. PLoS ONE.

[25] Geenfield N. (2006). Using circular dichroism spectra to estimate protein secondary structure. Nat. Protoc..

[26] Allen S.C., Muirhead H. (1996). Refined three-dimensional structure of cat muscle (M1) pyruvate kinase at a resolution of 2.6 Å. Acta Crystallogr. Sect. D Biol. Crystallogr..

[27] Larsen T.M., Laughlin L.T., Holden H.M., Rayment I., Reed G.H. (1994). Structure of Rabbit Muscle Pyruvate Kinase Complexed with Mn^2+^, K^+^, and Pyruvate. Biochemistry.

[28] Larsen T.M., Benning M.M., Wesenberg G.E., Rayment I., Reed G.H. (1997). Ligand-Induced Domain Movement in Pyruvate Kinase: Structure of the Enzyme from Rabbit Muscle with Mg^2+^, K^+^, and L-Phospholactate at 2.7 Å Resolution. Arch. Biochem. Biophys..

[29] Schormann N., Hayden K.L., Lee P., Banerjee S., Chattopadhyay D. (2019). An overview of structure, function, and regulation of pyruvate kinases. Protein Sci..

[30] Solomons T.G., Johnsen U., Schönheit P., Davies C. (2013). 3-Phosphoglycerate is an Allosteric Activator of Pyruvate Kinase from the Hyperthermophilic Archaeon *Pyrobaculum aerophilum*. Biochemistry.

[31] Abdelhamid Y., Brear P., Greenhalgh J., Chee X., Rahman T., Welch M. (2019). Evolutionary plasticity in the allosteric regulator binding site of pyruvate kinase isoform PykA from *Pseudomonas aeruginosa*. J. Biol. Chem..

[32] Roe D.R., Cheatham T.E. (2013). PTRAJ and CPPTRAJ: Software for Processing and Analysis of Molecular Dynamics Trajectory Data. J. Chem. Theory Comput..

[33] Ramírez-Silva L., Tuena de Gómez-Puyou M., Gómez-Puyou A. (1993). Water-Induced transitions in the K^+^ requirements for the activity of Pyruvate Kinase entrapped in Reverse Micelles. Biochemistry.

[34] Büchner T., Pleiderer G., Colowick S., Kaplan N. (1955). Pyruvate kinase from muscle. Methods in Enzymology.

[35] Shoemakers T.J.M., Visser G.J., Flik G., Theuvent P.R. (1992). CHELATOR: An improved method for computing metal ion concentrations in physiological solutions. Biotechniques.

[36] Doughtery T.M., Cleland W.W. (1985). pH studies of the chemical mechanism of rabbit muscle pyruvate kinase. 2. Physiological substrates and phosphoenol-αbutyrate. Biochemistry.

[37] Cottam G.L., Hollenberg P.F., Coon M.J. (1969). Subunit structure of rabbit muscle pyruvate kinase. J. Biol. Chem..

[38] DeLano W.L. (2004). Use of PYMOL as a communications tool for molecular science. Am. Chem. Soc..

[39] Ferrari A.J.R., Gozzo F.C., Martinez L. (2019). Statistical force-field for structural modeling using chemical cross-linking/mass spectrometry distance constraints. Bioinformatics.

[40] David A.C., Josh B., Betz R.M., Cerutti D.S., Cheatham T.E., Darden T.A., Duke R.E., Giese T.J., Gohlke H., Goetz A.W. (2015). AMBER 2015. https://ambermd.org/doc12/Amber15.pdf.

[41] Case D.A., Darden T.A., Cheatham T.E., Simmerling C.L., Wang J., Duke R.E., Luo R., Walker R.C., Zhang W., Merz K.M. (2012). AMBER 12. https://ambermd.org/doc12/Amber12.pdf.

[42] Case D.A., Cheatham T.E., Darden T., Gohlke H., Luo R., Merz K.M., Onufriev A., Simmerling C., Wang B., Woods R.J. (2005). The Amber biomolecular simulation programs. J. Comput. Chem..

[43] Walker R.C., Crowley M.F., Case D.A. (2008). The implementation of a fast and accurate QM/MM potential method in Amber. J. Comput. Chem..

[44] Tian C., Kasavajhala K., Belfon K.A.A., Raguette L., Huang H., Migues A.N., Bickel J., Wang Y., Pincay J., Wu Q. (2020). ff19SB: Amino-Acid-Specific Protein Backbone Parameters Trained against Quantum Mechanics Energy Surfaces in Solution. J. Chem. Theory Comput..

[45] Salomon-Ferrer R., Gotz A.W., Poole D., Le Grand S., Walker R.C. (2013). Routine Microsecond Molecular Dynamics Simulations with AMBER on GPUs. 2. Explicit Solvent Particle Mesh Ewald. J. Chem. Theory Comput..

[46] Kabsch W. (2010). Xds. Acta. Crystallogr. Sect. D Biol. Crystallogr..

[47] Evans P.R. (2006). Scaling and assessment of data quality. Acta. Crystallogr. Sect. D Biol. Crystallogr..

[48] Adams P.D., Afonine P.V., Bunkóczi G., Chen V.B., Davis I.W., Echols N., Headd J.J., Hung L.-W., Kapral G.J., Grosse-Kunstleve R.W. (2010). A comprehensive Python-based system for macromolecular structure solution. Acta Crystallogr. Sect. D Biol. Crystallogr..

[49] McCoy A.J., Grosse-Kunstleve R.W., Adams P.D., Winn M.D., Storoni L.C., Read R.J. (2007). Phaser crystallographic software. J. Appl. Crystallogr..

[50] Afonine P.V., Grosse-Kunstleve R.W., Echols N., Headd J.J., Moriarty N.W., Mustyakimov M., Terwilliger T.C., Urzhumtsev A., Zwart P.H., Adams P.D. (2012). Towards automated crystallographic structure refinement with phenix refine. Acta. Crystallogr. D. Biol. Crystallogr..

[51] Emsley P., Cowtan K. (2004). Coot: Model-building tools for molecular graphics. Acta Crystallogr. Sect. D Biol. Crystallogr..

[52] Zheng H., Cooper D.R., Porebski P.J., Shabalin I.G., Handing K.B., Minor W. (2017). CheckMyMetal: A macromolecular metal-binding validation tool. Acta Crystallogr. Sect. D Biol. Crystallogr..

